# Iatrogenic Pneumothorax Complicating Airway Management of Post-diphtheritic Tracheal Stenosis in a Pediatric Patient: A Case Report

**DOI:** 10.7759/cureus.102999

**Published:** 2026-02-04

**Authors:** Rati Prabha, Rajesh Raman, Abhishek Kumar, Syed M Raza, Kanchan Gupta

**Affiliations:** 1 Anesthesiology, King George's Medical University, Lucknow, IND; 2 Anesthesiology, Sanjay Gandhi Postgraduate Institute of Medical Sciences, Lucknow, IND

**Keywords:** diphtheria, emphysema subcutaneous, iatrogenic pneumothorax, pediatric patient, tracheal stenosis, tracheoplasty, tracheostomy

## Abstract

Diphtheria remains a significant cause of airway morbidity in under-immunized children. Emergency tracheostomy performed during the acute phase may lead to long-term complications such as tracheal stenosis, which poses major challenges during subsequent airway management. Iatrogenic pneumothorax is a rare but potentially fatal complication during emergency airway interventions. We report a case of a seven-year-old unvaccinated child with a history of diphtheria who underwent emergency tracheostomy followed by tracheoplasty for tracheal stenosis. Twenty days after decannulation, the child presented with acute respiratory failure due to post-tracheoplasty restenosis. Emergency airway management was attempted under spontaneous ventilation using inhalational anesthesia. Severe tracheal narrowing, scarring, and distorted anatomy resulted in difficult surgical access to the trachea. After securing the airway with a tracheostomy tube insertion, the child developed rapidly progressive subcutaneous emphysema and hemodynamic instability. Point-of-care lung ultrasound and chest radiography confirmed a large pneumothorax. Immediate needle thoracocentesis followed by chest tube insertion resulted in rapid clinical improvement. This case highlights the complexity of emergency airway management in children with post-diphtheria tracheal pathology. Early recognition of subcutaneous emphysema, prompt bedside imaging, and rapid intervention are critical to prevent catastrophic outcomes.

## Introduction

Despite the widespread availability of an effective vaccine, diphtheria continues to cause significant morbidity and mortality in under-immunized or unvaccinated pediatric populations [1,2]. It can cause airway obstruction, necessitating emergency tracheostomy in unvaccinated children. However, tracheostomy in children carries a high risk of complications, including tracheal injury, granulation tissue formation, and the long-term development of tracheal stenosis, particularly when performed in an emergent setting on an inflamed airway [3]. Managing a patient with post-tracheostomy or post-intubation tracheal stenosis is one of the most challenging scenarios in anesthesiology. When such a patient presents with acute respiratory failure, securing the airway is particularly difficult. We present a case of a seven-year-old child who developed a cascade of life-threatening complications, including severe hypercapnia and iatrogenic pneumothorax, during emergency airway management for impending respiratory arrest secondary to post-tracheoplasty restenosis.

## Case presentation

**Figure 1:**
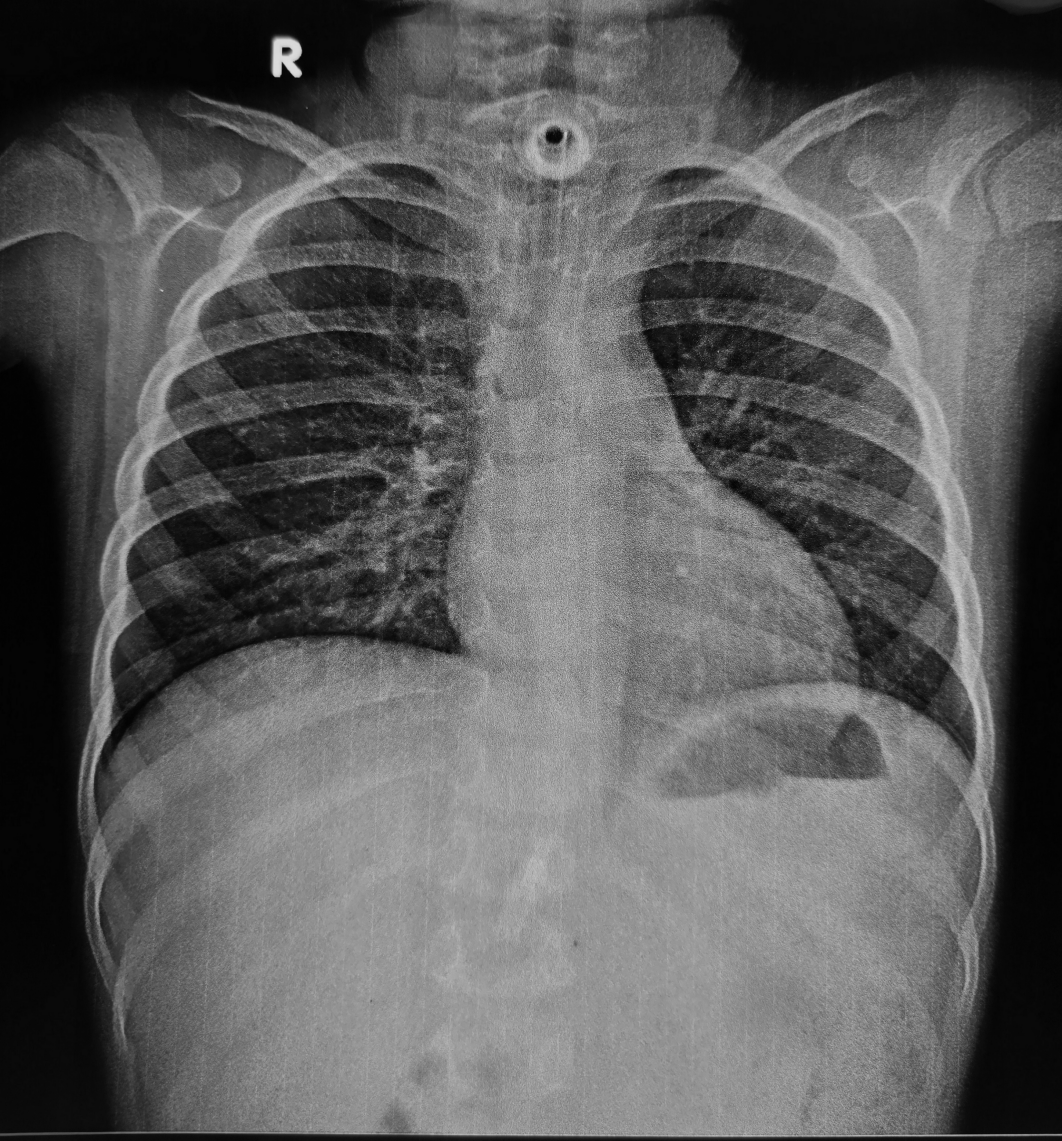
Chest X-ray of the patient before tracheoplasty.

A seven-year-old unvaccinated male presented with diphtheria and severe respiratory distress. Emergency tracheostomy was performed in the emergency department. His hospital course was complicated by a 10-day intensive care unit stay and difficult decannulation. The difficulty in decannulation was likely secondary to tracheal injury and subsequent narrowing of the airway. Neck radiography was performed, which confirmed tracheal narrowing. The patient was discharged home with a tracheostomy in place (Figure 1).

**Figure 2:**
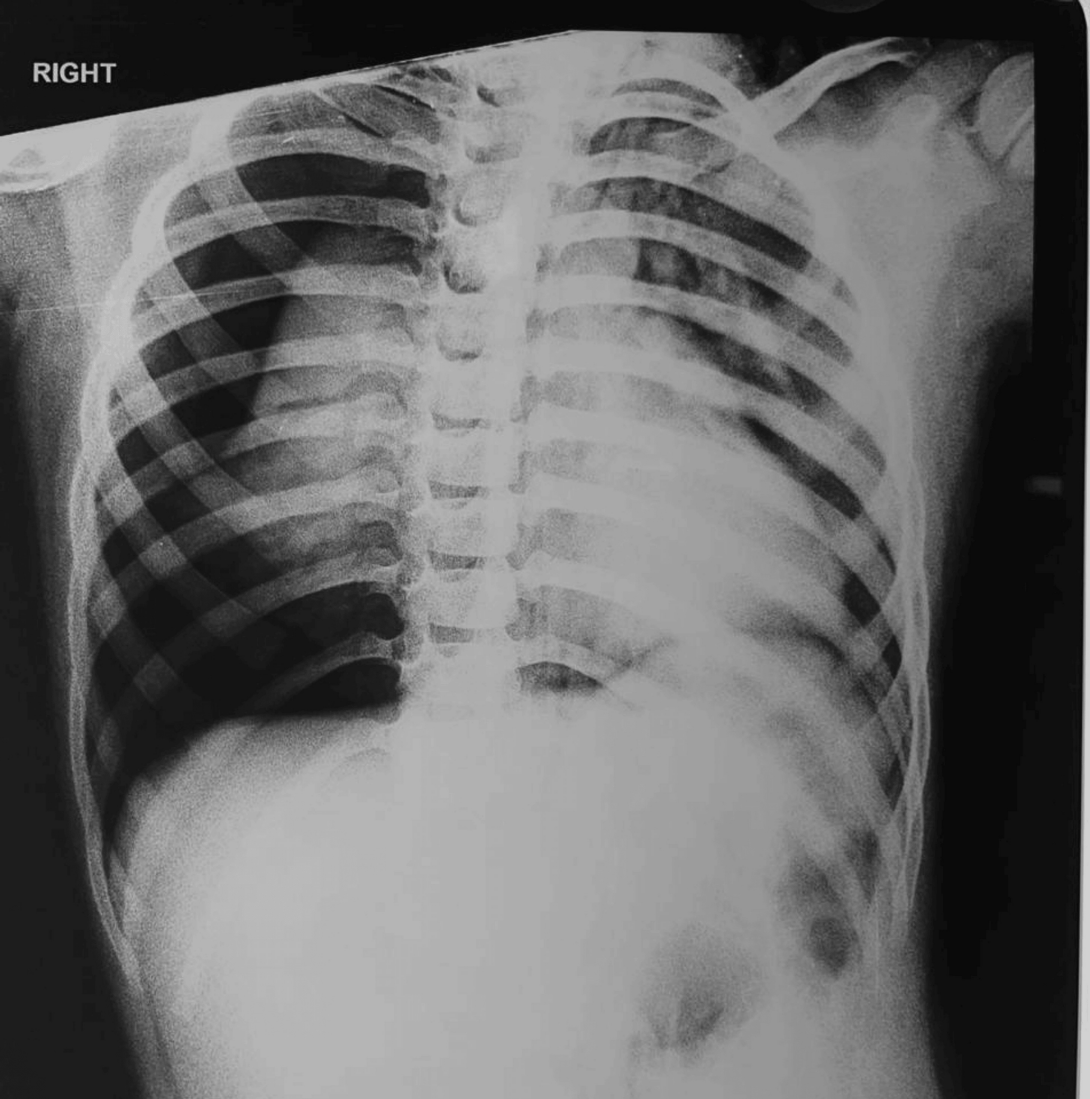
Intraoperative chest X-ray showing a large right-sided pneumothorax and a small left-sided pneumothorax.

Tracheoplasty was performed four months later for tracheal narrowing. After tracheoplasty, the child was successfully decannulated and discharged without a tracheostomy. Twenty days post-discharge, the patient developed acute, severe respiratory distress. On arrival, the patient was severely tachypneic with bilateral chest wall retraction and was agitated and disoriented, likely from hypercapnia. The patient had an oxygen saturation of 88% despite supplemental oxygen delivered via a non-rebreathing face mask. He was immediately transferred to the operating room, where 100% oxygen was administered through the anesthesia circuit along with low-dose sevoflurane (0.5-1 volume%) and intravenous fentanyl. Adequate sedation was achieved while maintaining spontaneous respiration. Direct laryngoscopy was subsequently performed, and the glottic opening was visualized; however, passage of even a 3.0-mm internal diameter endotracheal tube through the trachea was not possible. Local infiltration of 2% lignocaine was then administered in the midline of the neck in preparation for further airway intervention. Surgical exploration of the neck started with the immediate goal of identifying the trachea and inserting an airway in the distal trachea. The surgical field proved to be challenging due to post-operative scarring, fragile tissue, and oedema. Multiple attempts to locate the tracheal lumen were unsuccessful. The endotracheal tube was inserted in the false tract two times before the tracheal opening could be identified. A narrowed tracheal opening was identified, and a 3.0 mm internal diameter endotracheal tube was inserted. Ventilation was re-established through the endotracheal tube. End-tidal carbon dioxide (EtCO_2_) was 104 mm Hg at this point in time. To secure a more definitive airway, a guidewire was passed through the endotracheal tube. A 5.0 mm cuffed tracheostomy tube was then railroaded over the guidewire into the trachea, and ventilation was confirmed with bilateral chest rise and capnography. This resulted in adequate positive pressure ventilation of the patient. After this, intravenous vecuronium was administered for muscle relaxation. Ventilator settings were tidal volume 150 mL, respiratory rate 14 per minute, positive end-expiratory pressure of 3, and inspired oxygen concentration of 60% with an air-oxygen mixture. However, subcutaneous emphysema was noticed over the face of the patient. This was initially thought to be due to positive pressure ventilation through a false passage created during the multiple attempts to cannulate the trachea. The patient was kept under observation in the operation theater for monitoring, but the subcutaneous emphysema continued to increase rapidly. A point-of-care lung ultrasound was immediately performed. The scan revealed the absence of lung sliding and a barcode sign across the entire right hemithorax, indicating the presence of a significant pneumothorax. Absent lung sliding and barcode sign were also observed at the lower zone of the left hemithorax. An urgent portable chest X-ray was also done in the operating room. The chest X-ray confirmed a large right pneumothorax and a small pneumothorax on the left side (Figure 2).

At this point, the patient also developed hypotension (blood pressure 76/40 mm Hg) and tachycardia (heart rate 160 per minute). The peak airway pressure was also rising with an arterial oxygen saturation of 90%. The right hemithorax was immediately decompressed using needle thoracostomy, followed by insertion of an intercostal chest drain on the right side. The patient's hemodynamics and ventilation parameters improved markedly. He was then shifted to the intensive care unit for further management. The patient's subsequent intensive care unit course was uneventful, and the pneumothorax resolved on the second postoperative day. He was shifted from the intensive care unit to the ward in two days and discharged after two weeks on tracheostomy.

## Discussion

This case describes the complex challenges of pediatric airway management after diphtheria and tracheoplasty. Emergency airway management in such cases is challenging due to distorted anatomy and scarring. The primary anesthetic challenge was securing the airway in a patient with a narrowed trachea while avoiding complete respiratory collapse.

Maintenance of spontaneous ventilation during inhalational induction was a deliberate strategy to preserve airway patency and avoid the loss of respiratory drive that could result in a “cannot ventilate, cannot intubate” scenario. Maintaining spontaneous ventilation with a gentle sevoflurane induction helped the surgical team to locate the airway without the added pressure of an apneic patient. However, this approach carries inherent risks, including hypoventilation and hypercapnia, particularly when airway access is prolonged. The extremely elevated EtCO_2_ observed in this patient highlights the narrow therapeutic margin between maintaining spontaneous breathing and achieving adequate ventilation in complex pediatric airway scenarios.

The development of pneumothorax represents the most critical learning point from this case. The most probable mechanism was iatrogenic injury. The multiple attempts to cannulate the stenosed and scarred trachea likely created false passages in the para-tracheal soft tissues [4-6]. Subsequent application of positive pressure ventilation may have forced air through these disrupted tissue planes into the mediastinum and pleural cavity, leading to pneumothorax. Another mechanism could be damage to the lung apex or pleura during difficult and emergent surgical exploration for the trachea [5].

Another learning point from this case is subcutaneous emphysema as a red flag for potential airway disruption and pneumothorax [7]. Rapidly progressive subcutaneous emphysema after airway manipulation should raise suspicion for pneumothorax. Such patients should be closely monitored in the ICU and operating room. Maintenance of spontaneous respiration, readiness with smaller endotracheal tubes, early use of guidewires, and rapid transition to imaging modalities are also important. Moreover, multidisciplinary coordination among anesthesiologists, surgeons, and intensivists is essential for favorable outcomes of such patients.

This case report is novel in describing the occurrence of acute bilateral pneumothorax during emergency airway management in a pediatric patient with post-diphtheritic tracheal sequelae and prior tracheoplasty, a rare and high-risk clinical scenario. It highlights the early recognition of subcutaneous emphysema as a warning sign and demonstrates the utility of intraoperative point-of-care lung ultrasound for rapid diagnosis before radiographic confirmation. The report also provides practical insights into airway management strategies and rescue interventions in anatomically distorted pediatric airways.

## Conclusions

Children with airway pathology from diphtheria and subsequent tracheal reconstruction are at high risk for airway emergencies. Subcutaneous emphysema following airway intervention should immediately alert clinicians to possible pneumothorax. Prompt diagnosis with lung ultrasound and chest X-ray, and timely chest drain insertion can be lifesaving.
